# Assessment of the safety and feasibility of administering anti-pyretic therapy in critically ill adults: study protocol of a randomized trial

**DOI:** 10.1186/1756-0500-5-147

**Published:** 2012-03-16

**Authors:** Daniel J Niven, Caroline Léger, Paul Kubes, H Tom Stelfox, Kevin B Laupland

**Affiliations:** 1 Department of Critical Care Medicine, University of Calgary, and Alberta Health Services, Calgary, Alberta, Canada; 2 Office of the Associate Dean of Research, University of Calgary, Calgary, Alberta, Canada; 3 Department of Physiology and Pharmacology, University of Calgary, Calgary, Alberta, Canada; 4 Department of Medicine, University of Calgary, and Alberta Health Services, Calgary, Alberta, Canada; 5 Department of Community Health Sciences, University of Calgary, Calgary, Alberta, Canada

## Abstract

**Background:**

Fever is one of the most commonly observed abnormal signs in patients with critical illness. However, there is a paucity of evidence to guide the management of febrile patients without acute brain injury and little is known about the biologic response to treatment of fever. As such, observational studies suggest that the treatment of fever is inconsistent. This pilot clinical trial will assess the safety and feasibility of treating febrile critically ill adult patients with an aggressive versus a permissive temperature control strategy. The biologic response to these two different temperature control strategies will also be assessed through analysis of a panel of inflammatory mediators.

**Findings:**

The study population will include febrile adult patients admitted to one of two general medical-surgical intensive care units (ICUs) in Calgary, Alberta, Canada. Patients will be randomized to either an aggressive or permissive fever treatment strategy. The aggressive group will receive acetaminophen 650 mg enterally every 6 hours upon reaching a temperature ≥ 38.3°C and external cooling will be initiated for temperatures ≥ 39.5°C, whereas the permissive group will receive acetaminophen 650 mg every 6 hours upon reaching a temperature ≥ 40.0°C and external cooling for temperatures ≥ 40.5°C. The study will take place over 12 months with the goal of enrolling 120 patients. The primary outcome will be 28-day mortality after study enrolment, with secondary outcomes that will include markers of feasibility (e.g. the enrolment rate, and the number of protocol violations), and levels of select inflammatory and anti-inflammatory mediators.

**Discussion:**

Results from this study will lead to a better understanding of the inflammatory effects of anti-pyretic therapy and will evaluate the feasibility of a future clinical trial to establish the best treatment of fever observed in nearly one half of patients admitted to adult ICUs.

**Trial Registration:**

ClinicalTrials.gov: NCT01173367

## Background

Fever, an adaptive response to physiologic stress, is one of the most commonly observed abnormal signs in patients with critical illness, occurring in almost 50% of patients admitted to intensive care units (ICUs) [1-3]. Fever has also been shown to be independently associated with mortality in patients admitted to ICUs [1,4]. In spite of this, there is a lack of robust data to guide the management of pyrexia. This frequently leaves the decision to treat elevated body temperature at the discretion of the bedside nurse [5,6]. This decision is further complicated by the contradictory nature of the current available molecular data regarding the initiation and progression of fever, which leads to difficulties in knowing which pathways may be involved and therefore which temperature lowering strategy should be employed.

A core body temperature ≥ 38.3°C is generally accepted to represent fever which may occur as a result of a number of infectious and non-infectious etiologies [3]. The gold standard for measuring core temperature is the thermistor on a pulmonary-artery catheter (PAC). Similarly accurate temperature recordings can be obtained from urinary bladder catheter thermistors, esophageal probes, and rectal thermometers [7]. However, these methods of temperature measurement are generally too expensive and invasive to use on all critically ill patients. Therefore the temperature of many ICU patients is assessed using more cost-effective, less invasive, albeit less accurate devices such as the infrared tympanic membrane and temporal artery thermometers [3].

Few studies have reported on the epidemiology of fever in general medical and surgical ICU patients [1,4,8,9]. Circiumaru and colleagues found that prolonged fever was associated with increased mortality (62.5% versus 29.6%, *p *< 0.0001), however this was not adjusted for illness severity, etiology or other potential confounders [8]. Peres Bota et al found that fever occurred in 139 of 493 adult patients admitted to their tertiary care ICU (28.2%) [9]. Fever was commonly present at ICU admission (76.3%) and was frequently infectious in origin (55%). Febrile patients had significantly increased mortality compared to patients with normothermia (35.3% versus 10.3%, *p *< 0.01), however this was not adjusted for potential confounders. Barie and colleagues also found fever to occur among 26% of 2,419 patients admitted to their surgical ICU [4]. Patients with fever had an increased overall mortality compared to the afebrile cohort (26.5% versus 6.5%, *p *< 0.0001) and peak temperature was independently associated with mortality (odds ratio (OR) 1.84 for each one-degree increase in temperature, 95% confidence interval (CI) 1.23 - 2.75). In the largest study looking at the epidemiology of fever in general medical and surgical ICU patients, Laupland et al found that the cumulative incidence of fever was 44% among 20,466 patients admitted to ICUs in Calgary, Alberta, Canada between 2000-2006 [10]. Compared to medical patients that did not develop a fever during ICU admission, medical patients who were afebrile at ICU admission and subsequently went on to develop high fever were at increased risk of death (adjusted OR1.91, 95% CI 1.36 - 2.70). The reasons for this increased risk of death are unclear, but may arise from enhanced understanding of the effects of treating or not treating fever on the pathways involved in propagating or abrogating fever.

The production of fever involves a complex series of reactions between exogenous and endogenous pyrogens. Knowledge of these mechanisms is important as it will help to guide clinical decisions regarding the management of patients with pyrexia. The most cited mechanism for the induction of infectious fever is derived from a lipopolysaccharide (LPS) model of Gram-negative bacterial sepsis. In this model, LPS travels to the liver Kupffer cell which results in the production of the complement component C5a [11,12]. C5a promotes the release of prostaglandins (PGE_2_) from these cells, which transmits the message via the vagus nerve to the nucleus tractus solitarius in the brainstem [11,13,14]. The signal then travels to the hypothalamus and triggers both an early and a delayed rise in temperature. The early temperature rise is a result of the depression of the firing of warm-sensitive neurons via an α_1_-adreno receptor (AR), PGE_2_-independent pathway [15]. Clinically, this correlates with chills and rigors [16]. The delayed temperature rise starts with an interaction between norepinephrine and an α_2_-AR that results in an increase in PGE_2 _production via the cyclooxygenase-2 enzyme (COX-2) [15]. This is associated with a more prolonged rise in core temperature than that of the PGE_2_-independent pathway and is responsible for the persistence of fever in the face of an infectious stimulus.

This delayed pathway is also the site of action of anti-pyretics such as acetaminophen [13,17]. The effect of temperature lowering agents on the levels of many endogenous inflammatory (e.g. interleukin (IL)-1β, IL-6, and tumour necrosis factor (TNF)-α) and anti-inflammatory molecules (e.g. IL-1 receptor antagonist (IL-1 RA), IL-10, and TNFα-binding protein (TNFα-BP)) is unclear. Knowledge of the biologic consequences of fever management is important because excessively elevated temperatures are associated with adverse cellular effects yet strategies that lower temperature may impair the ability of the host to clear an infection.

Unfortunately, there is a paucity of evidence to guide the treatment of fever in critically ill patients and strong arguments exist to justify treating and not treating elevated body temperatures. Studies arguing against temperature lowering strategies have shown that naturally hypothermic septic patients are at higher risk for death than patients who are able to generate a fever [8,9,18,19]. In addition, commonly employed anti-pyretics (non-steroidal anti-inflammatories (NSAIDs), and/or acetaminophen) are associated with a not insignificant risk of bleeding, and hepatic and renal toxicity [20]. Treatment of fever may also mask the presentation and diagnosis of severe underlying infections. This in turn may delay administration of appropriate antimicrobial therapy which is clearly associated with increased mortality [21,22]. On the other hand, temperatures sustained at extremely high levels (> 40°C) may worsen cerebral edema and precipitate multisystem organ failure [23]. It is generally accepted that these extreme temperatures be lowered using pharmacologic and/or external cooling methods. However there is no consensus opinion with regard to the management of patients whose temperature is between 38.3°C and 40.0°C.

There are relatively few randomized trials that assess the effects of anti-pyretic therapy on critically ill adults without acute neurological injury. With the exception of one trial [24], most of these studies are limited by small sample size [25-28] or an inability to perform a true randomization of interventions [27]. Bernard et al randomized 455 septic patients with fever or hypothermia and at least one organ failure to receive either 10 mg/kg of ibuprofen every six hours for eight doses or placebo [24]. Unfortunately there was no difference in the primary outcome of 30-day mortality (37% for ibuprofen versus 40% for placebo); however this may have been due to a bias introduced by the differential use of acetaminophen within the intervention and placebo groups respectively. Gozzoli et al randomly assigned 38 febrile patients with systemic inflammatory response syndrome (SIRS) admitted to their surgical ICU to receive either external cooling or no anti-pyretic therapy for a temperature ≥ 38.5°C [26]. This study also did not show an appreciable difference in any clinically significant outcome.

Finally, Schulman et al randomized 82 febrile patients admitted to a surgical ICU to receive either an aggressive or permissive temperature management strategy [25]. The aggressive treatment group received acetaminophen 650 mg every 6 hours when their temperature was above 38.5°C and a cooling blanket was added if the temperature exceeded 39.5°C. The permissive treatment group did not receive treatment until their temperature exceeded 40.0°C at which point this group received the aggressive treatment protocol. This study was stopped early due to a trend towards an increased number of deaths in the aggressive group (seven versus one in the permissive group, *p *= 0.06). This may have been due to several factors, including the fact that patients required an antecedent ICU length of stay of 72 hours to be included in the study. This may have selected for patients with more severe nosocomial illnesses such as nosocomial pneumonia and bloodstream infection. These authors also did not report the proportion of patients who experienced cooling blanket intolerances and how this was managed (i.e. use of sedatives, neuromuscular blockers, or meperidine). An increased use of these drugs may help to explain the increased mortality observed in the aggressive fever treatment group. Finally, this study was not designed to detect a difference in mortality and this unexpected finding needs to be confirmed in a larger trial.

Given the lack of robust data to guide the management of temperature among febrile ICU patients, treatment with anti-pyretic agents is inconsistent. This is supported by the results of two studies that retrospectively looked at anti-pyretic prescribing practices in hospitalized patients [5,6]. Isaacs and colleagues found that 50% of patients admitted to their academic institution between 1986 - 1987 received an anti-pyretic order and 86% of anti-pyretic orders were written to be given "as needed" at the discretion of the bedside nurse. More recently, we found that 79 of 100 non-neurologically injured febrile critically ill adults received pharmacologic and/or physical anti-pyretic therapy. Few patients receiving this therapy had an explicit order to guide this management strategy (5/79). Though these two publications are separated by more than 20 years, the results are very similar and suggest that inconsistencies in fever management are likely the standard of care.

As it is unclear how to approach fever in the general ICU patient without acute brain injury (neurologically intact) or acute myocardial infarction, this pilot, randomized clinical trial will assess the safety and feasibility of treating febrile critically ill patients with an aggressive as compared to a permissive temperature management strategy. This study will also examine the effect of anti-pyretic therapy on consumption of anti-microbials, and the incidence of nosocomial infection, as well as the effect of anti-pyretic treatment of fever on markers of inflammation. This may lead to a better understanding of the inflammatory effects of anti-pyretic therapy and will lay the groundwork for a future clinical trial that may be expected to have a major influence on the treatment of nearly one half of patients admitted to adult ICUs.

## Methods

### Study design and participants

This will be a parallel, randomized clinical trial with 1:1 group allocation to either an aggressive or permissive fever treatment strategy. For the purposes of this study, the term fever treatment will refer to the use of anti-pyretic medications and/or physical cooling, whereas the term fever management will refer to the selection of appropriate investigations, the use of anti-microbials, and anti-pyretic therapies. The study protocol has been approved by the Conjoint Health Research Ethics Board (CHREB) at the University of Calgary and is registered (http://Clinicaltrials.gov identifier NCT01173367). The recruitment of study participants will take place in the Peter Lougheed Centre (PLC) ICU and the Foothills Medical Centre (FMC) ICU. The PLC ICU is a 16-bed multi-system ICU that admits a wide range of medical and surgical patients and is the non-cardiac vascular surgery referral centre for southern Alberta. The FMC ICU is a 25-bed unit that serves as the regional trauma and neurosurgical referral center, but also admits a wide range of medical and surgical patients. Eligibility criteria will be:

### Inclusion Criteria

• Age ≥ 18 years old

• Fever (two consecutive measurements ≥ 38.3°C at least 2 hours apart or a single temperature measurement ≥ 39.5°C)

• Admission to ICU with an expected length-of-stay ≥ 48 hours

• Attending physician approval

### Exclusion Criteria

• Admission to ICU for support for a specific procedure (e.g. endoscopy, acute dialysis, bronchoscopy)

• Acute brain injury due to any etiology

• Acute myocardial ischemia

• Documented hepatitis (alanine aminotransferase [ALT] more than twice the upper limit of normal), or chronic hepatic failure (defined by evidence of cirrhosis on available imaging or known varices, ascites, hepatic encephalopathy, hepatorenal syndrome, and/or hepatocellular carcinoma)

• Hyperthermia syndromes (malignant hyperthermia, heat stroke, neuroleptic malignant syndrome, serotonin syndrome, or endocrine causes including thyrotoxicosis, pheochromocytoma, and adrenal crisis)

• Refractory shock with lactic acidosis > 4 mmol/L (at the time of screening for study enrolment) despite supportive therapy

• Requirement for use of acetaminophen or non-steroidal anti-inflammatory drugs for indications other than treatment of fever

• Receipt of anti-pyretic pharmacotherapy within 6-hours of expected study enrolment (650 mg acetaminophen, 800 mg ibuprofen, or 325 mg acetylsalicylic acid)

• Contraindications to esophageal temperature monitoring

• Pregnancy (all women of child-bearing potential will have a pregnancy test performed)

• Time from onset of fever in the ICU to study enrolment > 12 hours (due to the need to assess the timing of expression of various inflammatory mediators)

Given the time constraints associated with timely measurement of inflammatory markers, the CHREB approved a waiver of initial consent for enrolment into this study. As such, if the patient or their SDM is not available to discuss consent, the patient will be enrolled into the study and consent obtained subsequent to initiation of the study protocol. The goal will be to obtain formal consent within 24-hours of study enrolment. If consent is unable to be obtained within 24-hours after enrolment, the patient will be excluded from further study participation. Patients and their SDM will be able to withdraw consent at any point during the study.

### Randomization and blinding

The randomization scheme will be developed using the technique of simple block randomization. This scheme will be generated and maintained by one of the co-principal investigators not directly involved with patient enrolment (H.T.S.). Allocation will be concealed using the consecutively, numbered, sealed opaque envelope technique. As such it will not be possible for the treating clinicians, or other study investigators to know the treatment group assignment prior to randomization. Envelopes will not be opened until the patient's name is recorded in the study log.

Due to cost and logistic constraints, patients, nurses, and the attending ICU team will not be blinded to study treatment assignment. However, lab personnel will be blinded to study group assignment, and all safety outcomes will be objectively reviewed in a blinded fashion by two intensivists not involved with patient enrolment.

### Temperature management protocol

The initial temperature measurement that meets febrile criteria will be obtained by the temporal artery method. Recent data has shown that temporal artery thermometers become inaccurate (by nearly 1°C) at the extremes of temperature [29]. Other more accurate, but somewhat invasive methods of temperature measurement include continuous bladder, esophageal, and rectal thermometers. As it will not be feasible to monitor all ICU patients with one of these continuous temperature monitors, the temporal artery method is a more cost-effective means of identifying patients for this study. Once a patient is enrolled in this study, their core temperature will be monitored using a continuous esophageal temperature probe until resolution of fever (defined as no fever for 3 consecutive days) or removal of their endotracheal tube.

Patients that meet enrolment criteria will be randomly allocated to the permissive or aggressive fever treatment group. Study patients will remain in their assigned group for the duration of their stay in the ICU. Patients assigned to the aggressive fever treatment protocol will receive acetaminophen 650 mg enterally every 6 hours for a temperature ≥ 38.3°C and external cooling will be initiated for temperatures ≥ 39.5°C. External cooling will consist of cool face cloths, ice packs, room fans, and a decrease in the ambient room temperature. Cooling blankets will be added at the discretion of the attending physician. Acetaminophen and external cooling will be discontinued once core temperature is less than 38.3°C and 39.5°C respectively. Patients assigned to the permissive treatment strategy will not receive anti-pyretic therapy until their temperature reaches 40.0°C at which point they will receive acetaminophen 650 mg every 6 hours. External cooling will be initiated for temperatures ≥ 40.5°C (as per interventions listed for aggressive group). Similar to the aggressive group, acetaminophen and external cooling will be discontinued once core temperature is less than 40.0°C and 40.5°C respectively. If at any time during this study, a patient's temperature reaches 41.0°C, regardless of group assignment, the methods of cooling will be left to the discretion of the attending physician until core body temperature is less than 40.5°C, at which point they will be managed according to that of their respective treatment arm. If at any point a patient, SDM or attending physician decides that ongoing participation in the study is not in the patient's best interest, the patient may be removed from the study.

### Laboratory studies

The plasma levels of 19 mediators relevant to inflammatory and/or pyrexia mechanisms will be examined (Table 1). Plasma will be isolated upon study admission and at 12, 24, and 48-hours after the start of the treatment. Venous or arterial blood will be collected from an existing intravascular line into a BD citrated plasma tube, gently inverted several times and centrifuged at 1700 × g for 10 minutes at 4°C. The plasma will then be collected, taking all necessary measures not to disturb the pellet, and aliquoted (250 ul each) into appropriately labelled cryopreservation tubes. The aliquots will be stored at -80°C until further analysis.

**Table 1:** Inflammatory mediators of interest

Mediator	Role in Pyrexia or Inflammation	Reference
IL-1β	Potent endogenous pyrogen	[30]

IL-1RA	Endogenous anti-pyretic cytokine	[31]

Il-4	Key mediator of humoral and adaptive immunity	[32]

IL-6	Potent pro-inflammatory cytokine and pyrogen	[30]

IL-8	Neutrophil chemotactic factor	[33]

IL-10	Endogenous anti-pyretic cytokine	[34]

IL-17	Potent pro-inflammatory mediator	[35]

IL-18	Amplifies the innate immune response	[36]

IFN-γ	Endogenous pyrogen	[30]

TNF-α	Endogenous pyrogen and ubiquitous inflammatory mediator	[37]

RANTES	Chemotactic factor for T-cells, dendritic cells, and NK cells	[38]

MIP-1α	Leukocyte chemotactic factor	[39]

GRO-α	Neutrophil chemotactic factor	[40]

TRAIL	Involved in inducing cellular apoptosis	[41]

sTNF-RI	Involved in mediating the apoptotic effects of TNF-α	[42]

sTNF-RII	Involved in modulating T-cell proliferation	[42]

HSPs (60, 70, 90)	Mediators of an adaptive response to cellular stress	[43]

Abbreviations: *IL-1β*, interleukin-1β; *IL-1RA*, interleukin-1 receptor antagonist; *IL-4*, interleukin-4; *IL-6*, interleukin-6; *IL-8*, interleukin-8; *IL-10*, interleukin-10; *IL-17*, interleukin-17; *IL-18*, interleukin 18; *IFN- γ*, interferon- γ; *TNF-α*, tumor necrosis factor-α; *RANTES*, Regulated upon Activation, Normal T-cell Expressed, and Secreted; *MIP-1α*, macrophage inflammatory protein-1α; *GRO-α*, growth-related oncogene-α; *TRAIL*, tumor necrosis factor-related apoptosis-inducing ligand; *sTNF-R1*, soluble tumor necrosis factor-α receptor-I; *sTNF-RII*, soluble tumor necrosis factor-α receptor-II; *HSPs*, heat shock proteins; *NK*, natural killer

The levels of the inflammatory mediators outlined in Table 1 will be determined by either Luminex technology or an enzyme-linked immunosorbent assay (ELISA). The Luminex equipment allows for measurement of several analytes at the same time using the x-MAP technology. This technique combines the specificity and reproducibility of the ELISA technique with the multiplexing capacity of the fluorescent-bead immunoassays, thus maximizing the utilization of the sample. Indeed, this technique requires as little as 100 μl total of plasma per patient for all the mediators described below. This technique is relatively novel but has been fully validated [44]. Furthermore, this multiplexing technology minimizes both the inter-assay variability and the time and cost required to perform these measurements. Finally, Bio-Plex Manager Software version 6.0 will be used for the analysis of the results as it automatically draws the standard curves and infers the concentration of each samples, limiting the possibility of human errors.

Two panels of commercially available antibody coupled beads will be selected to detect the following mediators. Panel A will be used to measure IL-1β, IL-1RA, Il-4, IL-8, IL-10, IL-17, IFN-γ, TNF-α, IL-6, RANTES, MIP-1α, sTNF-RI and sTNF-RII, while panel B will be used to measure IL-18, GRO-α and TRAIL. Two panels are required due to buffer incompatibilities. Each of these panels will be assayed following the manufacturer's instructions and read using a Luminex 200 instrument (Luminex, Texas, USA). The data will be analyzed using Bio-Plex Manager Software version 6.0.

As there is no commercially available Luminex-compatible bead to measure HSP60, HSP70 and HSP90 these mediators will be measured by a standard commercially available ELISA kit. Each mediator will be analyzed following the manufacturer's protocol provided with the kit. The data will be acquired using a PerkinElmer Victor X4 plate reader and analyzed with the WorkOut 2.5 software.

### Outcome Measures and Data Collection

Clinical data will be recorded from the time of enrolment until day 28 following enrolment and will be primarily obtained from the hospital electronic medical record (Quantitative Sentinel, GE-Marquette Medical Systems Inc. Milwaukee, WI, USA; Sunrise Clinical Manager version 5.7, Eclipsys Corporation; Web-based diagnostic imaging system, Agfa WEB1000 version 4.1 SP3) as previously described [1]. Survival at day 28 will be established by phone call follow-up for those patients discharged prior to this date. Patients will be followed for evidence of new nosocomial infection and these will be classified using standard Centres for Disease Prevention and Control criteria.

As this study is concerned with the safety and feasibility of the aforementioned temperature control regimen, the primary outcome will be mortality 28-days after study enrolment. Secondary outcomes of interest include markers of feasibility (i.e. enrolment rate [number of patients enrolled/number of eligible candidates], adherence of patients to assigned treatment regimen, and acceptance of the protocol by staff) and other markers of safety (nosocomial infection rate, evidence of myocardial ischemia, and/or hepatocellular inflammation). Other secondary outcomes include the total number of days of anti-microbials used, number of microbiologic and radiologic tests, and changes in the levels of the previously mentioned inflammatory mediators.

### Statistical analyses

All statistical analyses will be performed using Stata version 11.2 (Stata Corp, College Station, TX). Data will be analyzed according to the intention-to-treat method. Prior to description or statistical testing, all continuous variables will be asssessed for underlying distribution using histograms. Normally distributed variables will be reported as means ± standard deviations (SD) and skewed variables as medians with inter-quartile ranges (IQR). Mean and median values for clinical data will be compared using the Student's *t*-test or the K-sample equality of medians test. Similarly, differences in proportions among categorical data will be assessed using Fisher's exact test for pair-wise comparisons and the chi^2 ^test for multiple groups. The unadjusted primary outcome of 28-day mortality will be analyzed using Fisher's exact test. Logistic regression will be employed to provide an adjusted estimate of the odds of 28-day mortality. A priori defined variables to incorporate into this model include APACHE II score, admission diagnostic classification, and whether the fever is infectious in etiology. Other variables to include in the model will be those associated with a *p*-value ≤ 0.1 in the univariate analyses. Differences in the levels of the aforementioned panel of inflammatory mediators will be analyzed using univariate statistics appropriate for dependent data, namely a paired *t*-test for data with a normal distribution, or the Wilcoxon signed rank test for skewed data.

As this is a pilot study, a formal sample size calculation was not performed. However, an estimate of the projected recruitment over one-year was calculated based on previous work in this study's health region [1]. In the previously described study by Laupland et al, 5,753 patients were found to have fever among all patients admitted to four ICUs in the Calgary Health Region from 2000 - 2006 [1]. The PLC ICU and FMC ICU comprise roughly 63% of those ICU beds. This translates into an approximate 3,624 febrile patients over the seven years of that study, which equates to 43 patients per month with fever. Using a conservative estimate of capturing 25% of all potentially eligible patients, this study is expected to enroll 10 patients per month which will result in a total of 120 patients at the end of one year of recruitment. As this is a pilot study, the authors feel that this is an adequate number of patients to evaluate the safety and feasibility outcomes of interest. Thus the enrolment phase of this study is projected to require one-year.

## Competing interests

The authors declare that they have no competing interests.

## Authors' contributions

KBL conceived the research question and is the principal investigator for this study. DJN, KBL, and HTS drafted the clinical aspect of the study protocol. CL, and PK drafted the laboratory section of the study protocol and will be responsible for the analysis of the panel of inflammatory mediators. DJN was responsible for drafting this paper and all authors read, provided important revisions and approved the final version of the manuscript.
